# Traditional definition of healthcare-associated influenza underestimates cases associated with other healthcare exposures in a population-based surveillance system

**DOI:** 10.1017/ice.2023.64

**Published:** 2023-11

**Authors:** Erin B. Gettler, H. Keipp Talbot, Yuwei Zhu, Danielle Ndi, Edward Mitchel, Tiffanie M. Markus, William Schaffner, Bryan Harris, Thomas R. Talbot

**Affiliations:** 1 Division of Infectious Diseases, Department of Medicine, Duke University Medical Center, Durham, North Carolina; 2 Department of Health Policy, Vanderbilt University Medical Center, Nashville, Tennessee; 3 Division of Infectious Diseases, Department of Medicine, Vanderbilt University Medical Center, Nashville, Tennessee; 4 Department of Biostatistics, Vanderbilt University Medical Center, Nashville, Tennessee

## Abstract

**Objective:**

To provide comprehensive population-level estimates of the burden of healthcare-associated influenza.

**Design:**

Retrospective cross-sectional study.

**Setting:**

US Influenza Hospitalization Surveillance Network (FluSurv-NET) during 2012–2013 through 2018–2019 influenza seasons.

**Patients:**

Laboratory-confirmed influenza-related hospitalizations in an 8-county catchment area in Tennessee.

**Methods:**

The incidence of healthcare-associated influenza was determined using the traditional definition (ie, positive influenza test after hospital day 3) in addition to often underrecognized cases associated with recent post-acute care facility admission or a recent acute care hospitalization for a noninfluenza illness in the preceding 7 days.

**Results:**

Among the 5,904 laboratory-confirmed influenza-related hospitalizations, 147 (2.5%) had traditionally defined healthcare-associated influenza. When we included patients with a positive influenza test obtained in the first 3 days of hospitalization and who were either transferred to the hospital directly from a post-acute care facility or who were recently discharged from an acute care facility for a noninfluenza illness in the preceding 7 days, we identified an additional 1,031 cases (17.5% of all influenza-related hospitalizations).

**Conclusions:**

Including influenza cases associated with preadmission healthcare exposures with traditionally defined cases resulted in an 8-fold higher incidence of healthcare-associated influenza. These results emphasize the importance of capturing other healthcare exposures that may serve as the initial site of viral transmission to provide more comprehensive estimates of the burden of healthcare-associated influenza and to inform improved infection prevention strategies.

Accounting for an estimated 140,000–710,000 hospitalizations and 12,000–52,000 deaths in the United States annually since 2010,^1^ influenza is a significant cause of morbidity and mortality. Transmission of influenza to patients has occurred in a variety of healthcare settings,^2–6^ often as the result of infected healthcare personnel,^7^ and it is associated with increased length of stay and poor outcomes.^8^ Despite this impact, comprehensive estimates of the incidence of healthcare-associated influenza are limited. The most commonly used method to capture the burden of this important nosocomial infection defines healthcare-associated influenza as an influenza case diagnosed after day 3 of hospitalization,^2,9,10^ which ignores other preadmission healthcare exposures. Most published reports also only describe the experience of outbreaks at single institutions^2,8^ or the epidemiology of pandemic strains.^3,11^ Lack of standardized surveillance definitions and variation in clinician-driven testing inhibit comparability between these published studies^12^ and likely underestimate the number of healthcare-associated influenza hospitalizations.

Of the few published population-level estimates in the United States, the proportion of healthcare-associated influenza ranged from 0.4% to 2.8% of patients admitted with influenza.^9,10^ Although this frequency was relatively low, Jhung et al^9^ and Cummings et al^10^ found that patients with healthcare-associated influenza were more likely to have severe outcomes, including the need for ICU-level care, mechanical ventilation, and longer hospital stays.^9,10^ These studies, however, omit important preadmission healthcare exposures (eg, residence in a post-acute care facility, recent admission to an acute care facility, or key ambulatory healthcare visits) that may have served as the initial location of viral transmission.^13^ Limited to cases identified using the traditional definition of healthcare-associated influenza with test positivity and symptom onset after day 3 of hospitalization, these studies likely underestimated the overall burden of healthcare-associated influenza.

To perform a more comprehensive assessment of healthcare-associated influenza that includes preadmission healthcare exposures, we used data from the US Influenza Hospitalization Surveillance Network (FluSurv-NET) to evaluate the population-level burden of healthcare-associated influenza over 7 influenza seasons. The incidence of healthcare-associated influenza using the traditional definition was compared to that calculated with the addition of cases associated with recent post-acute care facility admission or a recent acute care hospitalization for a noninfluenza illness to better understand the frequency of other healthcare exposures that result in hospitalization for influenza.

## Methods

FluSurv-NET is a population-based, multicenter surveillance system that collects data on laboratory-confirmed influenza-associated hospitalizations in pediatric and adult patients in acute care hospitals within predefined counties in the 14 states participating in the Emerging Infections Program (EIP).^14,15^ The EIP is a collaborative network organized by the Centers for Disease Control and Prevention in partnership with state health departments and academic medical centers.^16^ Within FluSurv-NET, persons of any age residing within the defined catchment area admitted between October and April of every year with a positive influenza test collected within 14 days prior to or during the hospitalization are included. For each identified case, medical history, clinical course, medical interventions, and outcomes are obtained through medical chart abstraction performed by trained personnel. In this study, persons meeting the FluSurv-NET case definition and admitted between 2012 and 2019 and residing in an 8-county catchment area in middle Tennessee (ie, Cheatham, Davidson, Dickson, Robertson, Rutherford, Sumner, Williamson, and Wilson counties) were included. Laboratory confirmation was defined as a positive influenza test by molecular assay, rapid antigen testing, or viral culture.

**Fig. 1.**
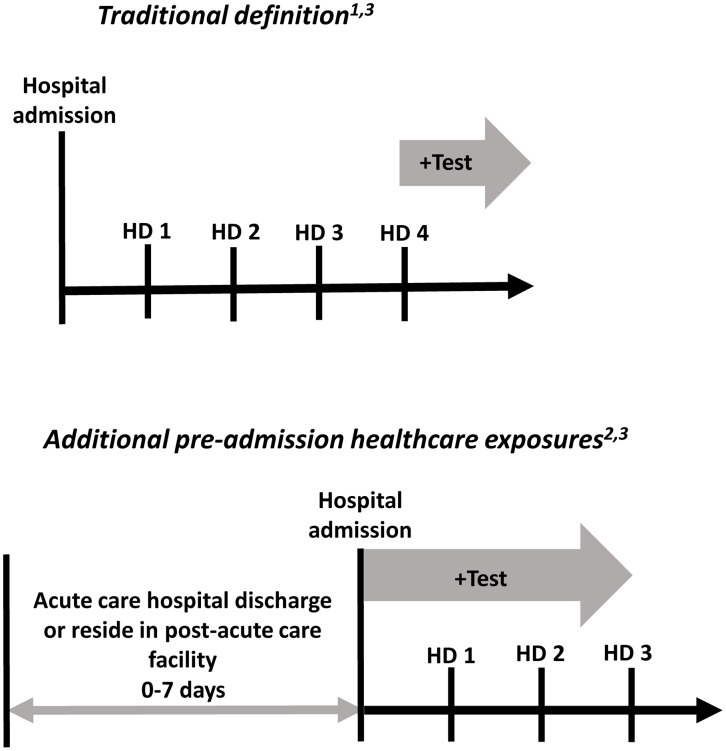
Traditional definition of healthcare-associated influenza and additional preadmission healthcare exposures.^1^Influenza-related hospitalizations with a positive test after day 3 were included in the traditional definition.^2^ Influenza-related hospitalizations with a positive test in the first 3 days of admission among patients directly transferred from a post-acute care facility or discharged from a non-influenza acute care hospitalization in the 7 days preceding the index admission were included in the additional preadmission healthcare exposure category.^3^ Cases meeting the traditional definition and those captured by the addition of the preadmission healthcare exposures were included in the expanded definition of healthcare-associated influenza. Note. HD, hospital day.

The “traditional” definition of healthcare-associated influenza was defined as cases that occurred in persons with a positive influenza test after day 3 of the index hospitalization.^2,9,10^ To include other preadmission healthcare settings in which influenza virus exposure and transmission may have occurred, we identified persons with a positive influenza test collected in the first 3 days of hospitalization who either (1) were transferred from a post-acute care facility, or (2) were discharged from an acute care facility for a noninfluenza illness in the 7 days preceding the index influenza admission (Fig. 1). These “additional healthcare exposures” combined with the cases meeting the “traditional” definition collectively represent the more comprehensive “expanded” definition of healthcare-associated influenza. Cases transferred directly from skilled nursing facilities, inpatient rehabilitation facilities, long-term acute care hospitals, inpatient hospice, and mental health facilities were included. Recent acute care hospital discharges were ascertained from FluSurv-NET and the Tennessee Department of Health Hospital Discharge Data System. Cases with only outpatient encounters in the days preceding admission could not be captured in the surveillance process and were therefore not included in the expanded definition.

Cases described as healthcare-associated influenza by either the traditional or expanded definitions were expressed as a percentage of the total laboratory-confirmed influenza-related hospitalizations by annual influenza season and overall. Additionally, the proportion of positive cases attributed to each type of healthcare exposure (ie, index hospitalization, associated with post-acute care transfer, or associated with recent acute care admission) were calculated. We calculated unadjusted incidence rates per 100,000 population stratified by type of healthcare exposure by year and overall using the number of cases meeting the definitional criteria as the numerator and the population within the predefined catchment area, as recorded by the National Center for Health Statistics’ vintage bridged-race postcensal population estimates,^17^ as the denominator. Additionally, a simple Poisson regression model was conducted to evaluate the association between cases and healthcare exposure using the traditional definition as the reference. Incidence rate ratios (IRRs) have been reported with their 95% confidence intervals (CI). Analyses were conducted in Stata version 16 software (StataCorp, College Station, TX). This study was approved by the Vanderbilt University Medical Center and Tennessee Department of Health Institutional Review Boards.

## Results

Among the 5,904 laboratory-confirmed influenza-related hospitalizations from 2012 through 2019 in the surveillance catchment area, most patients (52%) were aged ≥65 years (median age, 66 years; IQR, 50–79), were non-Hispanic White (73%), and had 1 or more underlying medical condition (91% of adults) (Table 1). In the total cohort, nearly 20% of patients admitted with influenza required ICU-level care and had a median hospital length of stay of 3 days (IQR, 2–6 days).

**Table 1.** Characteristics of Patients with an Influenza-Related Hospitalization, FluSurv-NET, 2012–2019

Characteristic	All Influenza Hospitalizations (Community and Healthcare-Associated) (N = 5,904), No. (%)	Healthcare-Associated Influenza Using Traditional Definition (N = 147), No. (%)	Healthcare-Associated Influenza Among Patients with a Preadmission Healthcare Exposure (N = 1,031), No. (%)	*P* Value^a^
**Sex**				.041
Male	2,615 (44)	74 (50)	427 (41)	
Female	3,289 (56)	73 (50)	604 (59)	
**Age group**				.293
0–17 y	430 (7)	9 (6)	36 (4)	
18–49 y	1,021 (17)	29 (20)	170 (17)	
50–64 y	1,402 (24)	26 (18)	205 (20)	
≥65 y	3,050 (52)	83 (56)	619 (60)	
Age, median y (IQR)	66 (50–79)	67 (49–81)	71 (55–83)	
**Race/Ethnicity^b^**				.008
American Indian/Alaska Native	3 (<1)	0	0	
Asian/Pacific Islander	71 (1)	2 (1)	4 (<1)	
Black	1237 (21)	35 (24)	179 (17)	
Latinx/Hispanic	95 (2)	1 (1)	12 (1)	
Multiracial	8 (<1)	1 (1)	1 (<1)	
White	4,309 (73)	100 (68)	813 (79)	
Unspecified, not reported	181 (3)	8 (5)	22 (2)	
**Month of positive influenza test**				.026
October	47 (1)	2 (1)	6 (1)	
November	148 (3)	2 (1)	21 (2)	
December	1536 (26)	52 (35)	239 (23)	
January	1659 (28)	41 (28)	337 (33)	
February	1321 (22)	22 (15)	242 (23)	
March	877 (15)	20 (14)	135 (13)	
April	316 (5)	8 (5)	51 (5)	
**Influenza type**				.519
Influenza A	4718 (80)	122 (83)	823 (80)	
Influenza B	1106 (19)	25 (17)	195 (19)	
Influenza A/B, type not specified	78 (1)	0	12 (1)	
Unknown	2 (<1)	0	1 (<1)	
Transferred from another hospital	147 (2)	7 (5)	55 (5)	.771
Children <18 y with ≥1 underlying medical condition	204 (47)	6 (67)	13 (36)	.097
Adults ≥ 18 y with ≥1 underlying medical condition	4,957 (91)	129 (93)	934 (94)	.695

a Healthcare-associated influenza among patients with pre-admission healthcare exposures was compared to healthcare-associated influenza as defined by the traditional definition using chi-square.

b Self-reported race and ethnicity as captured in the electronic medical record.

Of these influenza-related hospitalizations, 147 (2.5%) had traditionally defined healthcare-associated influenza. Relative to the total cohort, these patients had a higher proportion of ICU admission (32% vs 19%; *P* < .001) and a longer median length of stay (12 days vs 3 days, respectively).

**Fig. 2.**
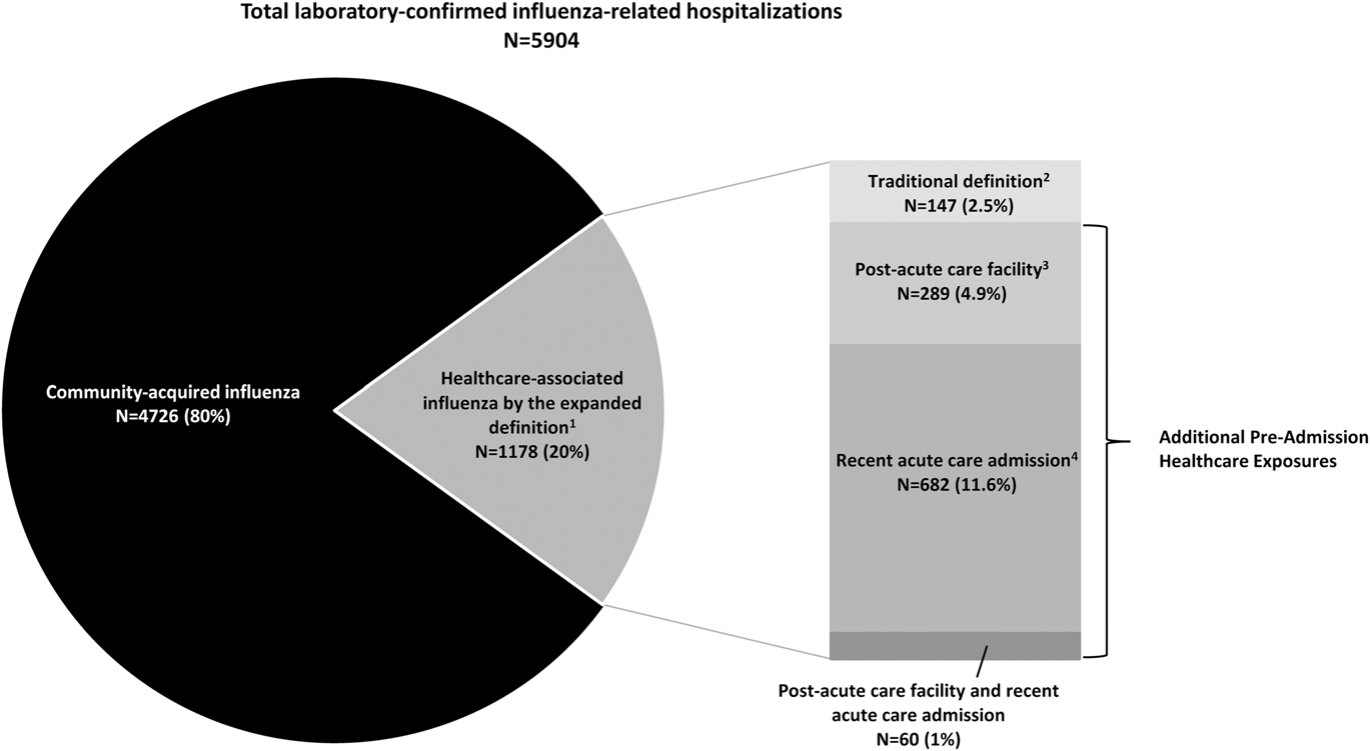
Proportion of influenza-related hospitalizations by type of healthcare exposure, FluSurv-NET, 2012–2019.^1^Cases meeting the traditional definition and those captured by the additional preadmission healthcare exposures were included in the expanded definition of healthcare-associated influenza.^2^Influenza-related hospitalizations with a positive test after hospital day 3 were included in the traditional definition.^3^Influenza-related hospitalizations with a positive test in the first 3 days of admission in patients directly transferred from a post-acute care facility.^4^Influenza-related hospitalizations with a positive test in the first 3 days of admission in patients discharged from a noninfluenza acute care hospitalization in the 7 days preceding index admission.

The inclusion of persons with a positive influenza test obtained in the first 3 days of hospitalization with a preadmission healthcare exposure identified an additional 1,031 cases (17.5% of all influenza-related hospitalizations). Of these additional cases, 289 (4.9%) were transferred from a post-acute care facility, 682 (11.6%) had a recent acute care hospital admission in the preceding 7 days, and 60 (1%) had both a recent acute care hospital admission and contact with a post-acute care facility (Fig. 2). Compared to those cases defined using only the traditional definition, cases defined by these additional healthcare exposures were more likely female (59% vs 50%; *P* = .041) and non-Hispanic White (79% vs 68%; *P* = .008). Within this group, the median length of stay more closely resembled that of the total cohort; however, similar to cases defined using only the traditional definition, the cohort identified with the additional healthcare exposures had a higher proportion of ICU admission than the total cohort of laboratory-confirmed influenza (27% vs 19%; *P* < .001).

**Fig. 3.**
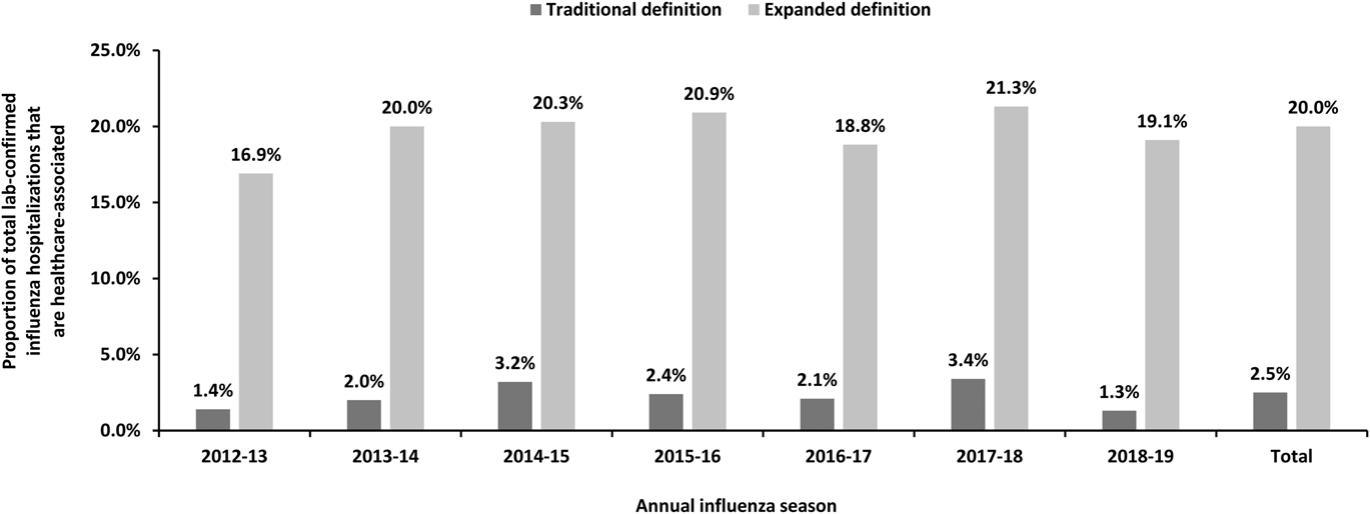
Proportion of influenza-related hospitalizations by traditional and expanded definitions of healthcare-associated influenza, FluSurv-NET, 2012–2019.

After including the additional healthcare exposures into the expanded definition, the proportion of laboratory-confirmed influenza-related hospitalizations that were healthcare-associated increased from 2.5% to 20%. The proportion of healthcare-associated influenza cases varied by each annual influenza season, ranging from 1.3% to 3.4% for cases defined using the traditional definition and 16.9% to 21.3% for cases defined using the expanded definition (Fig. 3).

**Fig. 4.**
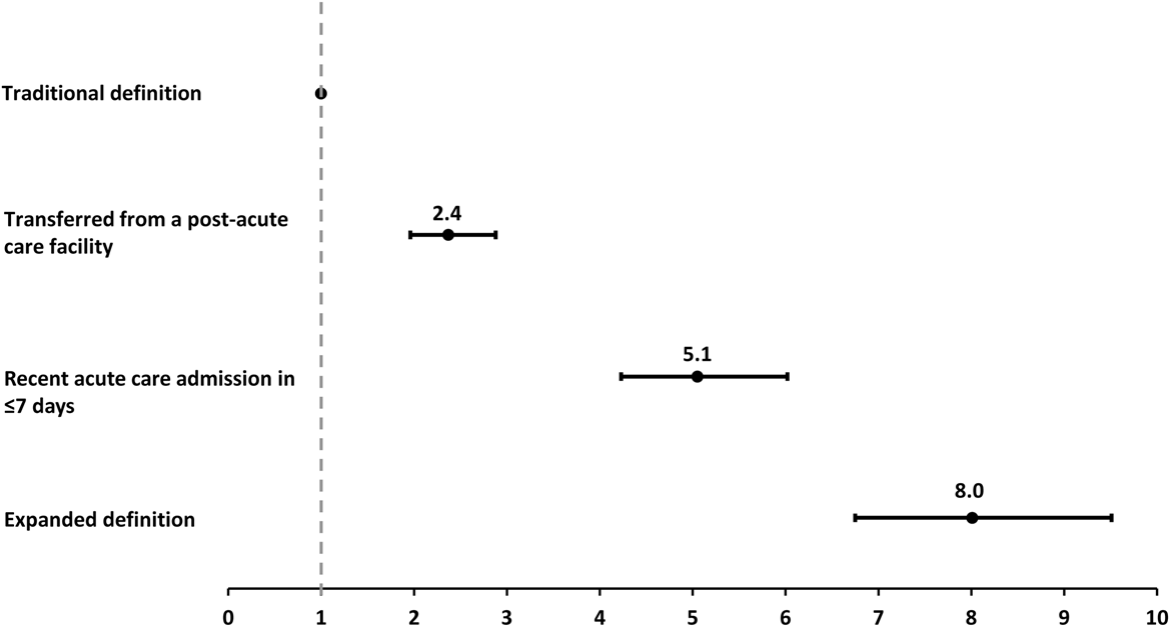
Incidence rate ratio of healthcare-associated influenza by type of preadmission healthcare exposure, FluSurv-NET, 2012–2019.

Among all influenza-related hospitalizations, the unadjusted rate of traditionally defined healthcare-associated influenza was 1.3 per 100,000 (with an annual seasonal range of 0.4–3.5, data not shown). This rate increased to 10.2 per 100,000 (range, 4.7–22.1) when the more comprehensive expanded definition of healthcare-associated influenza was used. Relative to the traditional definition, the incidence rate ratio of healthcare-associated influenza by the expanded definition was over 8-fold higher (8.0; 95% CI, 6.8–9.5) (Fig. 4).

## Discussion

Comprehensive estimates of the incidence of healthcare-associated influenza in the United States are limited. The majority of published studies describe outbreaks within single institutions,^2,3,8,11^ and the variability used to define healthcare-associated influenza limits comparison across studies.^12,18^ In addition, very few studies utilize a larger population-level surveillance to examine healthcare-associated influenza burden, and these reports were limited to traditionally defined cases with hospital-onset of symptoms or positive diagnostic testing after 3 days of hospitalization.^9,10,19^ The current study addresses several of these limitations. Using a population-based surveillance system of laboratory-confirmed hospitalized cases of influenza over 7 influenza seasons in middle Tennessee. Our findings further demonstrate that the traditional definition of hospitalized healthcare-associated influenza underestimates the potential true incidence of this important nosocomial infection by failing to include preadmission healthcare exposures.

These findings reinforce and augment those from a recently published study from the Canadian Nosocomial Infection Surveillance Program between 2006 and 2012. In their analysis, laboratory-confirmed influenza was considered in those patients with symptom onset within 96 hours after transfer from another facility or readmission <96 hours after discharge in addition to those patients with more traditional hospital-onset of symptoms. Including these additional healthcare exposures, the frequency of healthcare-associated influenza was much higher at 17.3% of all hospitalized influenza cases,^20^ similar to the findings of our study. Moreover, 60.5% of the cases were associated with a recent stay in a long-term care facility,^20^ emphasizing the need for more comprehensive influenza surveillance and implementation of infection prevention strategies in these non-acute care settings.

Outbreaks of influenza in long-term care facilities have been well described,^4,6^ yet relatively little is known about the degree to which outpatient or other healthcare settings contribute to the incidence of healthcare-associated influenza. In a retrospective cohort study among pediatric patients, a significant association was detected between outpatient clinic visits and the increased incidence of influenza-like illness (ILI) after a non-ILI encounter during periods of high respiratory virus transmission.^13^ Improved understanding of how these healthcare settings may contribute to healthcare-associated influenza has important implications for horizontal infection control practices and may generate novel ways to lessen viral spread. These settings often present unique challenges to the general application of infection prevention practices that differ from those used in inpatient facilities,^4,21,22^ but these settings provide care for a number of particularly vulnerable patients with a burgeoning aging population and shift in the delivery of increasingly complex subspecialty care to outpatient settings.

Standardized and more complete definitions of healthcare-associated influenza could also improve clinician awareness and early detection of influenza as patients hospitalized with influenza may not present with the common signs and symptoms associated with an influenza-like illness.^23^ Timely recognition leads to rapid implementation of respiratory isolation precautions and reduced subsequent nosocomial transmission. As the results of this study suggest, healthcare-associated influenza has been associated with adverse outcomes, including longer length of stay, as well as increased need for ICU-level care, mechanical ventilation, and mortality relative to community-acquired influenza.^19^

This study had several limitations. First, determination of an influenza-related hospitalization is dependent on clinician-driven testing. Variability in clinical practice and institutional testing protocols may have resulted in significant underrecognition of influenza burden.^24^ We did not apply any multipliers to the burden estimate to account for testing practices; therefore, these burden estimates are very conservative. Secondly, categorizing cases as community- or healthcare-associated based on the date of test positivity may lead to misclassification, particularly without chart review to assess for symptoms consistent with ILI before test positivity. Not all potential preadmission healthcare exposures (eg, dialysis, infusion clinic, and outpatient clinic visits) were captured in the expanded definition, possibly leading to misattribution of cases as community-associated if the actual viral transmission occurred in these healthcare settings. Additionally, only influenza cases that required hospitalization were captured. Notably, the delay between admission and the date of test positivity used in the traditional definition is conservative based on the median incubation period for influenza (ie, 1.4 and 0.6 days for influenza A and B, respectively),^25^ thereby increasing the specificity of our definition. For these reasons, these results still may underestimate the true burden of healthcare-associated influenza.^18^ Conversely, including cases that occurred within 7 days of discharge from an acute care facility may misrepresent some cases that were actually community acquired. The challenge in attributing a source of transmission of a case of influenza must be acknowledged, as one can rarely determine this with absolute confidence. Hence, the use of the term “healthcare associated” and not “healthcare acquired,” which provides the association that can direct infection prevention interventions that should reduce healthcare-associated virus transmission even with the inability to definitively attribute the source of transmission of a specific case. Lastly, while these study results support the need for comprehensive and systematic surveillance of healthcare-associated influenza, there are barriers to capturing these other exposures accurately as part of a routine surveillance program. Improved data collection and tracking in the electronic medical record could assist with this effort.

In summary, restricting surveillance of healthcare-associated influenza to only those cases possibly acquired during the current acute care admission underestimates the prevalence of this important yet underrecognized nosocomial infection. Standardization of definitions and surveillance methods could improve the understanding of influenza transmission and inform clinical practice and infection prevention strategies to mitigate healthcare-associated transmission across all types of healthcare facilities.
